# BRCA1 and BRCA2 mutations in Turkish breast/ovarian families and young breast cancer patients

**DOI:** 10.1054/bjoc.2000.1332

**Published:** 2000-08-17

**Authors:** H Yazici, O Bitisik, E Akisik, N Cabioglu, P Saip, M Muslumanoglu, G Glendon, E Bengisu, S Ozbilen, M Dincer, S Turkmen, I L Andrulis, N Dalay, H Ozcelik

**Affiliations:** Centre for Cancer Genetics, Department of Pathology and Laboratory Medicine, Samuel Lunenfeld Research Institute, Mount Sinai Hospital, Toronto, Canada; Basic Oncology Department, Istanbul University Oncology Institute; Breast Surgery Department, Istanbul Medical Faculty; Clinical Oncology Department, Istanbul University Oncology Institute; Gynecology Department, Istanbul Medical Faculty; Department of Biochemistry, Istanbul Samatya SSK Training Hospital, Istanbul, Turkey; Department of Laboratory Medicine and Pathobiology, Molecular and Medical Genetics, University of Toronto; Cancer Care Ontario, Toronto, Canada

**Keywords:** BRCA1, BRCA2, mutations, PTT, breast and ovarian cancer, Turkish population

## Abstract

To date, BRCA1 and BRCA2 mutations in breast and/or ovarian patients have not been characterized in the Turkish population. We investigated the presence of BRCA mutations in 53 individuals with a personal and family history of breast and/or ovarian cancer, and 52 individuals with a personal history of breast cancer diagnosed below age 50 without additional family history. We have identified 11 mutations (nine BRCA1 and two BRCA2) using combined techniques involving protein truncation test, direct sequencing and heteroduplex analysis. We found eight out of 53 patients (15.1%) with a family history to carry BRCA gene mutations (seven BRCA1 and one BRCA2). Of these, four were found in 43 families presenting only breast cancer histories, and four were found in families presenting ovarian cancer with or without breast cancer. We also demonstrated two BRCA1 and one BRCA2 mutations in three out of 52 (5.8%) early-onset breast cancer cases without additional family history. Three of nine BRCA1 and both BRCA2 mutations detected in this study were not reported previously. These mutations may be specific to the Turkish population. The BRCA1 5382insC mutation, specific to Ashkenazi and Russian populations, was found twice in our study group, representing a possible founder mutation in the Turkish population. © 2000 Cancer Research Campaign


British Journal of Cancer (2000) 83(6), 737–742
© 2000 Cancer Research Campaign
doi: 10.1054/ bjoc.2000.1332, available online at http://www.idealibrary.com on IDEAL®

### BRCA1 and BRCA2 mutations in Turkish breast/ovarian families and young breast cancer patients

**H Yazici[1,2], O Bitisik[2], E Akisik[2], N Cabioglu[3], P Saip[4], M Muslumanoglu[3], G Glendon[9], E Bengisu[5], S Ozbilen[4], M Dincer[4], S Turkmen[6], IL Andrulis[1,7–9], N Dalay[2] and H Ozcelik[1,7]**

[1]Centre for Cancer Genetics, Samuel Lunenfeld Research Institute, and Department of Pathology and Laboratory Medicine, Mount Sinai Hospital, Toronto, Canada; [2]Istanbul University Oncology Institute, Basic Oncology Department; [3]Istanbul Medical Faculty, Breast Surgery Department; [4]Istanbul University Oncology Institute, Clinical Oncology Department; [5]Istanbul Medical Faculty, Gynecology Department; [6]Istanbul Samatya SSK Training Hospital, Department of Biochemistry, Istanbul, Turkey; [7]Department of Laboratory Medicine and Pathobiology; [8]Molecular and Medical Genetics, University of Toronto; [9]Cancer Care Ontario, Toronto, Canada

**Summary** To date, BRCA1 and BRCA2 mutations in breast and/or ovarian patients have not been characterized in the Turkish population. We investigated the presence of BRCA mutations in 53 individuals with a personal and family history of breast and/or ovarian cancer, and 52 individuals with a personal history of breast cancer diagnosed below age 50 without additional family history. We have identified 11 mutations (nine BRCA1 and two BRCA2) using combined techniques involving protein truncation test, direct sequencing and heteroduplex analysis. We found eight out of 53 patients (15.1%) with a family history to carry BRCA gene mutations (seven BRCA1 and one BRCA2). Of these, four were found in 43 families presenting only breast cancer histories, and four were found in families presenting ovarian cancer with or without breast cancer. We also demonstrated two BRCA1 and one BRCA2 mutations in three out of 52 (5.8%) early-onset breast cancer cases without additional family history. Three of nine BRCA1 and both BRCA2 mutations detected in this study were not reported previously. These mutations may be specific to the Turkish population. The BRCA1 5382insC mutation, specific to Ashkenazi and Russian populations, was found twice in our study group, representing a possible founder mutation in the Turkish population. © 2000 Cancer Research Campaign

**Keywords**: BRCA1; BRCA2; mutations; PTT; breast and ovarian cancer; Turkish population

Germline mutations in breast cancer susceptibility genes BRCA1 and BRCA2 account for the majority of families with hereditary breast and ovarian cancers (Easton et al, 1993; Miki et al, 1994; Wooster et al, 1995; Tavtigian et al, 1996). Carriers of these mutations have an increased life-time risk of developing breast and ovarian cancers (Ford et al, 1994; Berman et al, 1996; Phelan et al, 1996). Both genes are large and multi-exonic: the coding region of BRCA1 contains 5592 base pairs distributed over 22 coding exons and BRCA2 contains 10 254 base pairs of coding sequence within 26 exons (Miki et al, 1994; Tavtigian et al, 1996). To date, more than 500 distinct sequence alterations distributed throughout the coding region have been described in each gene (Couch and Weber, 1996; Breast Cancer Information Core, 1999). Therefore, BRCA mutation analysis is hampered by the large size of the genes and by the diversity of multiple distinct mutations.

According to Turkish statistics, the breast cancer incidence in Turkey in 1994 was 6.14 per 100 000. In that year breast cancer constituted 23.04% of all cancers, representing the most common type of cancer in Turkish women (Turkish Ministry of Health, 1994). Founder mutations of both BRCA1 and BRCA2 have been described for several ethnic or geographically isolated populations (Szabo and King, 1997). The majority of hereditary breast cancer families of Ashkenazi Jewish ancestry are due to a restricted number of BRCA mutations (185delAG and 5382insC in BRCA1 and 6174delT in BRCA2) (Abeliovich et al, 1997; Levy-Lahad et al, 1997). In 24% of Icelandic women with familial breast cancer, a single mutation in the BRCA2 gene (999del5) is observed (Johannesdottir et al, 1996). The presence of founder mutations in a population simplifies the identification of those with inherited susceptibility to breast and ovarian cancer. This will facilitate both the development of hereditary cancer research and the clinical application of genetic testing for the population studied.

Studies on the BRCA1 and BRCA2 genes in the Turkish population have not been published and the spectrum of mutations is not known. In this study we report our observations of the frequencies and types of BRCA1 and BRCA2 mutations in a group of Turkish breast and/or ovarian cancer patients with and without a family history of cancer.

*Received 27 September 1999*
*Revised 3 April 2000*
*Accepted 11 May 2000*

*Correspondence to:* H Ozcelik

### MATERIALS AND METHODS

### Peripheral blood specimens

We recruited 53 patients with a family history of breast and/or ovarian cancers, and 52 early-onset breast cancer cases below age 50 without a family history of cancer. These patients were referred to the Istanbul University Oncology Institute and the Department of Surgery of the Istanbul Medical Faculty. Families were selected from consecutive referrals to the above-listed hospitals meeting specific family-history criteria (either multiple cases of breast and or ovarian cancer or the presence of an individual with a young age at diagnosis). Blood samples were obtained from all patients and genomic DNA was extracted using the phenol/chloroform extraction method.

### Protein truncation test

All samples were initially analysed for exon 11 mutations of both BRCA1 and BRCA2 using the protein truncation test (PTT) as described previously (Ozcelik et al, 1996; 1997). The PTT analysis was restricted to the analysis of exon 11 of each gene since cellular RNA samples were not available from these patients. Exon 10 of BRCA2 was also analysed by using protein truncation test as described (Hakansson et al, 1997).

### Sequencing analysis

Sequencing analysis of BRCA1 gene was carried out by automated sequencing (ABI, Foster City, CA, USA). The primer sequence information was obtained from the primer database (Breast Cancer Information Core, 1999) and the sequencing was carried out according to the company protocols.

### Heteroduplex analysis

Heteroduplex analysis was carried out for exons 2, 14 and 20 of the BRCA1 gene. PCR products from exons 2 (160 bp), 14 (236 bp) and exon 20 (120 bp) were amplified using the following primers: Exon 2, forward 5′-CTTCGCGTTGAAGAAGTAC-3′ and reverse 5′-GTCTTTTCTTCCCTAGTATGT-3′; Exon 14, forward 5′-CTAACCTGAATTATCACTATCA-3′ and reverse 5′-AAGATGTCAGATACCACAGC-3′; and exon 20, forward 5′-TGGAAGAAACCACCAAGGTC- 3′, reverse 5′-GGGGAG TGGAATACAGAGTGGT-3′.

PCR reactions for all three exons were carried out in a 50 µl reaction volume containing 1 × PCR buffer (10 mM Tris-HCl (pH 8.3), 50 mM KCl, 0.01% Gelatin), 3 mM $MgCl_2$, 112.5 mM of each deoxyribonucleoside triphosphate (dNTP), 10 pmol of each primer, 1 U of AmpliTaq DNA polymerase (Perkin Elmer/Cetus, Foster City, CA, USA) and 200 ng of genomic DNA. The PCR reaction was carried out for 30 cycles. The thermal cycling conditions were 94°C for 30 s, 55°C for 30 s and 72°C for 1 min.

Heteroduplex analyses were carried out using 1 × MDE (FMC BioProducts, Maine, USA) gel system for all three exons. The electrophoresis was performed at 70 V overnight in 0.6 × TBE (89 mM Tris-base, 89 mM Boric Acid, 2 mM EDTA pH 8.0) buffer. The results were visualized under UV-transilluminator.

### RESULTS

### BRCA1 and BRCA2 mutational analysis

We investigated the presence of BRCA1 and BRCA2 mutations in 53 patients with a personal and family history of breast and/or ovarian cancers, and 52 breast cancer patients below age 50. All samples were initially analysed for exon 11 mutations in both BRCA1 and BRCA2 and exon 10 in BRCA2 using the protein truncation test. The PTT analysis was restricted to the exons noted above, as cellular RNA samples were not available from these patients. We identified six truncating mutations in exons 11 (four BRCA1 and two BRCA2) of the 105 patients studied (Figure 1A, B) No mutations were found in exon 10 of BRCA2 (Table 1).

The remaining exons of the BRCA1 gene were also sequenced in a subset of 24 patients. Families were selected from three categories of family history: families with multiple breast cancers, families with multiple breast and ovarian cancers, and families with only early-onset breast cancer. The first consecutive 14, five and five patients were selected from the above categories, respectively. We identified two additional mutations outside exon 11 of BRCA1: insertion of a ‘C’ in exon 20 (5382insC), and deletion of a ‘G’ at the first nucleotide of intron 14 (IVS-14+1delG). The latter mutation resulted in the skipping of exon 14 during splicing, leading to a frameshift in the reading frame, and premature termination of the protein.

Heteroduplex analysis was performed to specifically investigate the presence of BRCA1 5382insC and IVS-14+1delG mutations in the rest of the samples not sequenced for the entire BRCA1 coding region. An additional 5382insC mutation was identified in a patient (Figure 2A). We did not detect additional IVS-14+1delG mutations, however the exon 14 heteroduplex analysis revealed a novel 4508delG BRCA1 mutation (Figure 2B). Since the 5382insC mutation is one of the common Ashkenazi Jewish mutations, we have also looked for the exon 2, 185delAG mutation in our families. Although no del185AG mutations were identified, the exon 2 heteroduplex analysis did detect a single 185insA mutation (Figure 2C).

### Distribution of mutations in groups

Using the approach described above, a total of 11 mutations (nine BRCA1 and two BRCA2) were identified in the 105 samples studied (Table 1). We found 15.1% (8/53) of Turkish patients with a family history of breast and/or ovarian cancer to have BRCA1 and BRCA2 mutations (Table 2). Three (two BRCA1 and one BRCA2) mutations were observed in families with three or more breast cancer cases with at least two in a first-degree relationship. Only one BRCA1 mutation was found in 34 families with two cases of breast cancer in a first or second-degree relationship. Four BRCA1 mutations were found in ten families with breast and ovarian cancer.

We also studied 52 breast cancer patients diagnosed between the ages of 20 and 50 years without additional cancer history. Among these 52, 28 cases were between ages 20 and 35, and 24 cases were between 36 and 50. The average age of this group was 34.46 ± 7.26. Three of the 52 (5.8%) were found to carry BRCA mutations (Table 2). Two of the mutations were in BRCA1 and one was in BRCA2. The patients carrying these mutations were 37 (BRCA1), 38 (BRCA1) and 40 (BRCA2) years old, respectively.

### DISCUSSION

In this study BRCA1 and BRCA2 mutations were investigated in Turkish breast and ovarian cancer patients using combined techniques. Among 53 cases with a family history of breast and/or ovarian cancers, we have detected seven BRCA1 and one BRCA2 mutations. The presence of a mutation was related to the strength of the family history of breast and ovarian cancer. Forty percent of families with ovarian cancer with or without family history of breast cancer and 33% of families with three or more breast cancer cases in a first or second-degree relationship were found to carry BRCA mutations (Table 2). However, only 3% of families with two cases of breast cancer in close relatives were found to harbour BRCA mutations. This experience is similar to other international groups attempting to find BRCA mutations in breast and ovarian cancer families (Couch et al, 1997; Ford et al, 1998).

Mutational analysis was also performed on 52 women with breast cancer diagnosed under the age of fifty, who had no

*© 2000 Cancer Research Campaign*

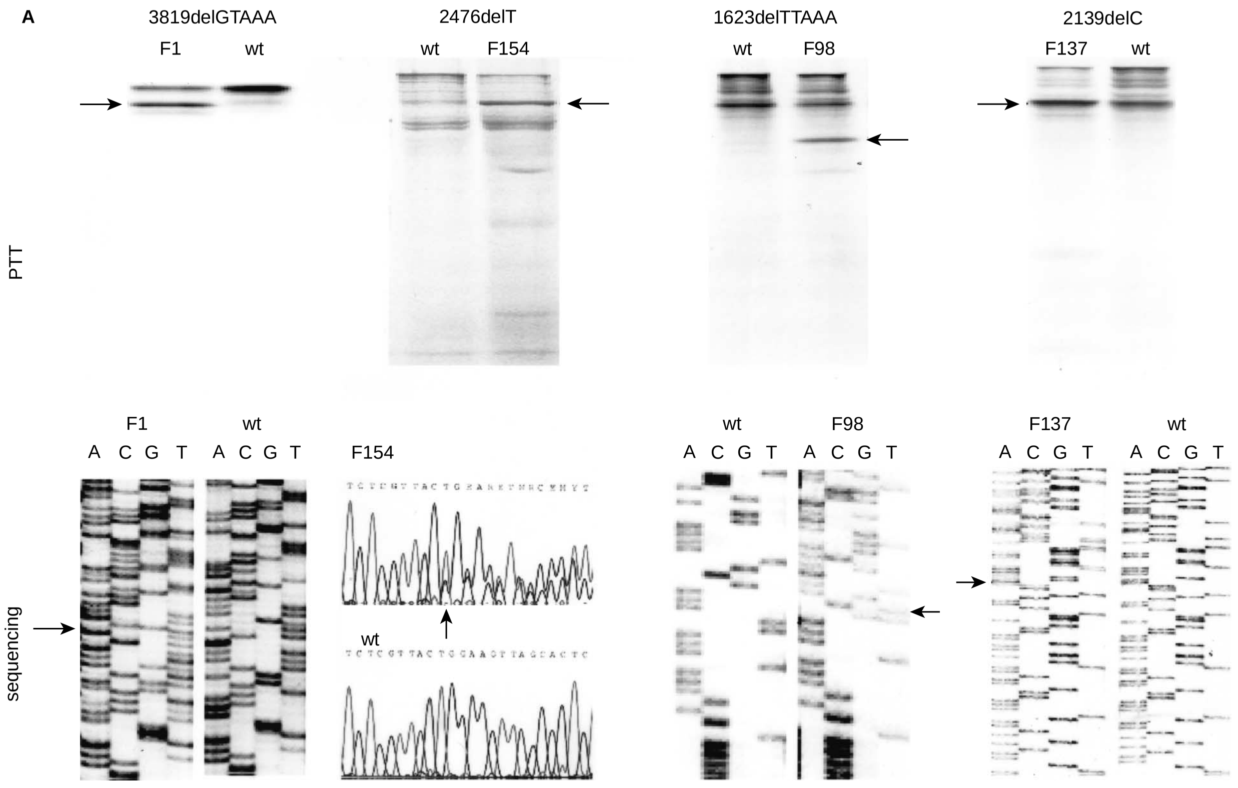

**Figure 1** (**A**) Identification of BRCA exon 11 mutations PTT and sequencing analysis of exon 11 of BRCA1. The top panel demonstrates the truncated PTT products (indicated by an arrow) in samples F1, F154, F98 and F137. On each gel wt represents a control case for the region of exon 11. Lower panel shows the sequencing result for each PTT-positive case (indicated by an arrow).

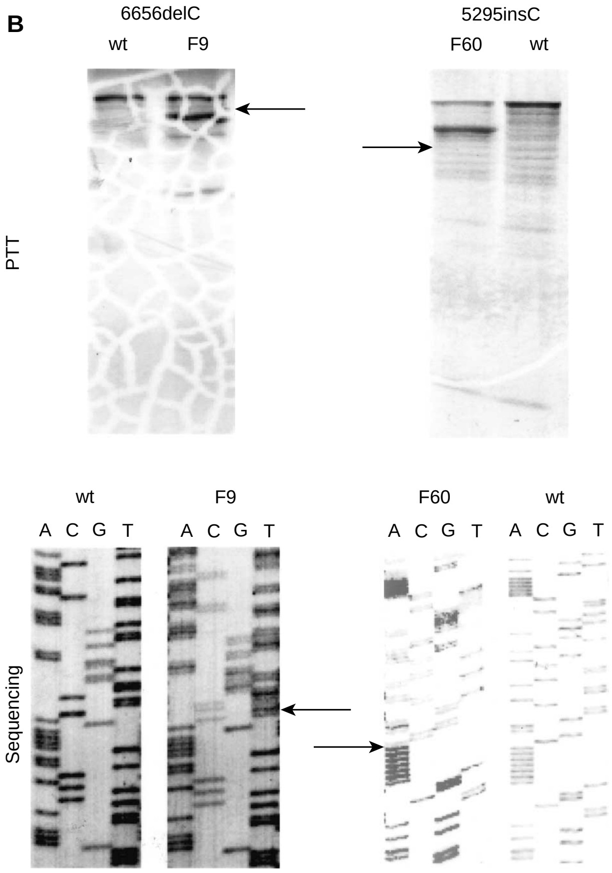

**Figure 1** (**B**) Identification of BRCA exon 11 mutations PTT and sequencing analysis of exon 11 of BRCA2. Top panel shows the two BRCA2 mutations detected in exon 11 of cases F9 and F60. Lower panel shows the actual location of mutations (indicated by an arrow).

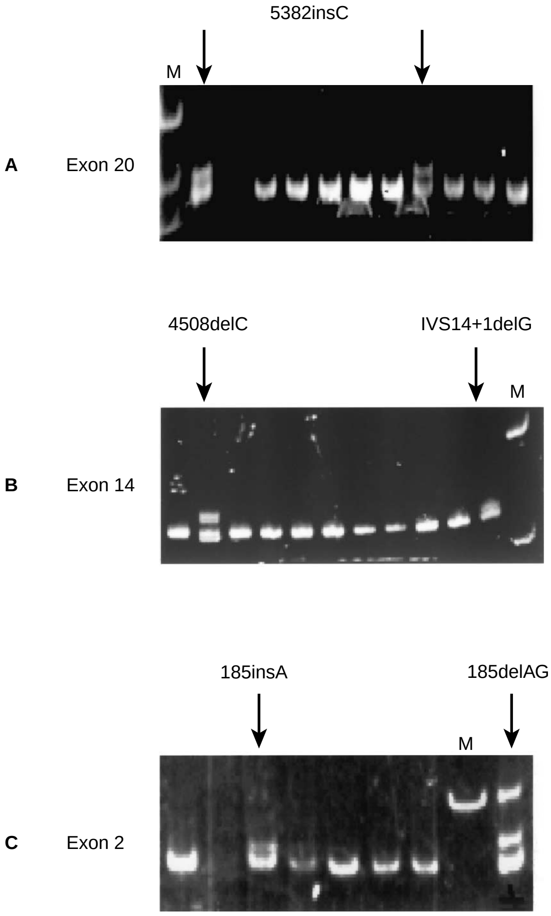

**Figure 2** Heteroduplex analysis of three exons of BRCA1. Gel electrophoresis for all exons are performed using MDE gel system. An arrow indicates positive cases and the mutation type is indicated on the top. (**A**) Heteroduplex analysis of exon 20. One of the cases with the 5382insC mutation represents a positive control. (**B**) Heteroduplex analysis of exon 14. The IVS-14+1delG mutation is used as a positive control. (**C**) Heteroduplex analysis of exon 2. The 185delAG mutation is used as a positive control. M = DNA marker identifiable family history of cancer. This approach allowed us to determine whether or not early age at diagnosis, in the absence of additional family history of cancer, is a determinant of inherited susceptibility. Three of 52 (5.8%) women in this group were found to carry BRCA mutations. Two BRCA1 mutations were observed in patients at the age of 37 and 38, respectively. The patient with the BRCA2 mutation had breast cancer diagnosed at age 40. No mutations were identified in 28 women diagnosed before the age of 35. However, the difference in the distribution of mutations between the two age-groups may not be significant due to the

**Table 1** BRCA1 and BRCA2 mutations detected in Turkish individuals

| Family No. | Type of Cancer | Exon | Mutation | Effect | Fhx | BIC |
|---|---|---|---|---|---|---|
| **BRCA1 mutations** | | | | | | |
| 98 | Breast | 11 | 1623delTTAAA | Frameshift | No | Yes |
| 137 | Breast | 11 | 2139delC | Frameshift | Yes | Yes |
| 1 | Breast | 11 | 3819delGTAAA | Frameshift | Yes | Yes |
| 154 | Ovary | 11 | 2476delT | Frameshift | Yes | No |
| 51 | Breast | 14 | 4508delC | Frameshift | Yes | No |
| 142 | Breast | Int 14 | IVS-14+1delG | Splice Error | Yes | No |
| 49 | Ovary | 20 | 5382insC | Frameshift | Yes | Yes |
| 65 | Breast | 20 | 5382insC | Frameshift | No | Yes |
| 147 | Breast | 2 | 185insA | Frameshift | Yes | Yes |
| **BRCA2 mutations** | | | | | | |
| 60 | Breast | 11 | 5295insA | Frameshift | No | No |
| 9 | Breast | 11 | 6656delC | Frameshift | Yes | No |

 © 2000 Cancer Research Campaign

**Table 2** Frequency of BRCA1 and BRCA2 mutations

| Phenotype | Number of cases/families | BRCA1 mutations | BRCA2 mutations | Ratio (%) |
|---|---|---|---|---|
| **Families** | | | | |
| 3 breast cancer cases with at least 2 in a first-degree relationship | 9 | 2 | 1 | 3/9 (33.0) |
| 2 breast cancer cases in a first or second-degree relationship | 34 | 1 | 0 | 1/34 (3.0) |
| 2 first-degree relatives with ovarian or breast and ovarian cancer | 10 | 4 | 0 | 4/10 (40.0) |
| **Total** | **53** | **7** | **1** | **8/53 (15.1)** |
| **Early-onset cases** | | | | |
| 20–35 years | 28 | – | – | 0/28 (0) |
| 36–50 years | 24 | 2 | 1 | 3/24 (12.5) |
| **Total** | **52** | **2** | **1** | **3/52 (5.8)** |

small sample size. The presence of BRCA mutations in a population of women with early-onset breast cancer, without a cancer family history, has also been documented in other studies (Langston et al, 1996; Southey et al, 1999). Our observations support the notion that individuals with early-onset breast cancer, without family history, should also be considered candidates at risk for inherited susceptibility to breast and ovarian cancer in the Turkish population.

Five mutations detected in this study have not previously been reported in the Breast Cancer Information Core Database (Table 1). The remaining mutations have been reported in individuals of German, Spanish, Dutch, Belgian, Russian and Jewish ethnic background (Breast Cancer Information Core, 1999). This suggests that the mutations detected in this population are influenced by the ethnic admixture and the geographical location of the population studied. Accordingly, genetic admixture of β-globin gene mutations between Turkey and other neighbouring countries has been already demonstrated (Tadmouri et al, 1998).

The only mutation detected twice in our study population was the BRCA1 5382insC mutation. The same type of BRCA1 mutation has been demonstrated to occur repeatedly in the Ashkenazi Jewish and Russian populations due to founder effect (Abeliovich et al, 1997; Gayther et al, 1997; Levy-Lahad et al, 1997; Csokay et al, 1999). Haplotype analysis would help to determine if these mutations found in our cohort were ancestrally related to those found in other populations.

Identification of BRCA mutations in a substantial proportion of our patients indicates that these genes play a role in the incidence of breast cancer in the Turkish population. Due to limitations we were unable to analyse the entire BRCA coding regions in all the patients. Exons 10 and 11 of BRCA2 and exon 11 of BRCA1 where analysed in all patients. These exons constitute approximately 60% of the coding regions of each gene. If a roughly uniform distribution of mutations throughout both genes exists in the Turkish population, we would estimate that 60% of protein-truncating mutations were detected by this approach. We have also sequenced a subset of cases for the BRCA1 gene and screened the remaining patients for specific BRCA1 mutations. Therefore, the BRCA1 analysis is more complete than that for BRCA2 in this cohort. A more complete analysis of the BRCA genes might yield an even greater proportion of families with mutations. Due to the selection of those at a high genetic risk in our cohort, our mutation frequency may not reflect that of the Turkish breast cancer population in general. However, this study demonstrates the presence of BRCA mutations in families with breast and ovarian cancer and should be considered an integral element in the management of disease risk in Turkey.

On the other hand, the families in this study were ascertained based on the presence of family history or early onset of disease. The proportion of mutations found in our study population is most likely an overestimate of the proportion of mutations in the general Turkish population of breast and ovarian cancer patients. A population-based ascertainment scheme would provide the most accurate estimate of this. However, the families in this study may more accurately represent the types of families that would request, and more likely benefit from, genetic testing in a research or clinically based setting.

This is the first study to report the types of BRCA mutations in the Turkish population. Our results suggest that BRCA1 and BRCA2 mutations are observed in a significant proportion of Turkish families with breast and/or ovarian cancer, and those with early onset of disease. Our study demonstrates the importance of the consideration of inherited predisposition to breast and ovarian cancer in the clinical management of breast cancer risk in Turkey.

### ACKNOWLEDGEMENTS

We thank Ontario Cancer Genetics Network, Cancer Care Ontario. This project was supported by the Istanbul University Research Fund (UP-3-190397), The State Planning Organization in Turkey (97K121700) and NATO (CRG960130).

### REFERENCES

Abeliovich D, Kaduri L, Lerer I, Weinberg N, Amir G, Sagi M, Zlotogora J, Heching N and Peretz T (1997) The founder mutations 185delAG and 5382insC in BRCA1 and 6174delT inBRCA2 appear in 60% of ovarian cancer and 30% of early-onset breast cancer patients among Ashkenazi women. *Am J Hum Genet* **60**: 505–514

Berman DB, Costalas J, Schultz DC, Grana G, Daly M and Godwin AK (1996) A common mutation in BRCA2 that predisposes to a variety of cancers is found in both Jewish Ashkenazi and non-Jewish individuals. *Cancer Res* **56**: 3409–3414

Breast Cancer Information Core (1999) *http://www.nhgri.nih.gov/Intramural_research/Lab_transfer/Bic/Member/index.html*

Couch FJ and Weber BL (1996) Mutations and polymorphisms in the familial early-onset breast cancer BRCA1 gene. Breast cancer Information Core. *Hum Mutat* **8**: 8–18

Couch FJ, DeShano ML, Blackwood MA, Calzone K, Stopfer J, Campeau L, Ganguly A, Rebbeck T and Weber BL (1997) BRCA1 mutations in women

*© 2000 Cancer Research Campaign*

attending clinics that evaluate the risk of breast cancer. *N Engl J Med* **336**: 1409–1415

Csokay B, Tihomirova L, Stengrevics A, Sinicka O and Olah E (1999) Strong founder effects in BRCA1 mutation carrier breast cancer patients from Latvia. *Hum Mutat* **14**: 92

Easton DF, Bishop DT, Ford D and Crockford GP, The Breast Cancer Linkage Consortium (1993) Genetic linkage analysis in familial breast and ovarian cancer: results from 214 families. *Am J Hum Genet* **52**: 678–701

Ford D, Easton DF, Bishop DT, Narod SA and Goldgar DE, Breast Cancer Linkage Consortium (1994) Risks of cancer in BRCA1-mutation carriers. *Lancet* **343**: 692–695

Ford D, Easton DF, Stratton M, Narod S, Goldgar D, Devilee P, Bishop DT, Weber B, Lenoir G, Chang-Claude J, Sobol H, Teare MD, Struewing J, Arason A, Scherneck S, Peto J, Rebbeck TR, Tonnin P, Neuhausen S, Barkardottir R, Eyfjord J, Lynch H, Ponder BA, Gayther SA, Birch JM, Lindblom A, Stoppa-Lyonnet D, Bignon Y, Borg A, Hamann U, Haites N, Scott RJ, Maugard CM, Vasen H, Seitz S, Cannon-Albright, Schofield A and Zelada-Hedman M, Breast Cancer Linkage Consortium (1998) Genetic heterogeneity and penetrance analysis of the BRCA1 and BRCA2 genes in breast cancer families. *Am J Hum Genet* **62**: 676–689

Friedman LS, Szabo CI, Ostermeyer EA, Dowd P, Butler L, Park T, Lee MK, Goode EL, Rowell SE and King MC (1995) Novel inherited mutations and variable expressivity of BRCA1 alleles, including the founder mutation 185delAG in Ashkenazi Jewish families. *Am J Hum Genet* **57**: 1284–1297

Gayther SA, Harrington P, Russell P, Kharkevich G, Garkavtseva RF and Ponder BA (1997) Frequently occurring germ-line mutations of the BRCA1 gene in ovarian cancer families from Russia. *Am J Hum Genet* **60**(5): 1239–1242

Hakansson S, Johannsson O, Johansson U, Sellberg G, Loman N, Gerdes AM, Holmberg E, Dahl N, Pandis N, Kristoffersson U, Olsson H and Borg A (1997) Moderate frequency of BRCA1 and BRCA2 germ-line mutations in Scandinavian familial breast cancer. *Am J Hum Genet* **60**(5): 1068–1078

Johannesdottir G, Gudmundsson J, Bergthorsson JT, Arason A, Agnarsson BA, Eiriksdottir G, Johannsson OT, Borg A, Ingvarsson S, Easton DF, Egilsson V and Barkardottir RB (1996) High prevalence of the 999del5 mutation in Icelandic breast and ovarian cancer patients. *Cancer Res* **56**: 3663–3665

Langston AA, Malone KE, Thompson JD, Daling JR and Ostrander EA (1996) BRCA1 mutations in a population-based sample of young women with breast cancer. *N Engl J Med* **334**(3): 137–142

Levy-Lahad E, Catane R, Eisenberg S, Kaufman B, Hornreich G, Lishinsky E, Shohat M, Weber BL, Beller U, Lahad A and Halle D (1997) Founder BRCA1 and BRCA2 mutations in Ashkenazi Jews in Israel: frequency and differential penetrance in ovarian cancer and in breast-ovarian cancer families. *Am J Hum Genet* **60**(5): 1059–1067

Neuhausen S, Gilewski T, Norton L, Tran T, McGuire P, Swensen J, Hampel H, Borgen P, Brown K, Skolnick M, Shattuck-Eidens D, Jhanwar S, Goldgar D and Offit K (1996) Recurrent BRCA2 6174delT mutations in Ashkenazi Jewish women affected by breast cancer. *Nat Genet* **13**: 126–128

Miki Y, Swensen J, Shattuck-Eidens D, Futreal PA, Harshman K, Tavigain S, Liu Q, Cochran C, Bennet LM, Ding W, Bell R, Rosenthal J, Hussey C, Tran T, McClure M, Frye C, Hattier T, Phelps R, Haugen-Strano A, Katcher H, Yakumo K, Gholami Z, Shaffer D, Stone S, Bayer S, Wray C, Bogden R, Dayananth P, Ward J, Tonin P, Narod S, Bristow PK, Norris FH, Helvering L, Morrison P, Rosteck P, Lai M, Barret C, Lewis C, Neuhausen S, Cannon-Albright L, Goldgar D, Wiseman R, Kamb A and Skolnick M (1994) A strong candidate for the breast and ovarian cancer susceptibility gene BRCA1. *Science* **266**: 66–71

Ozcelik H, Antebi Y, Cole DEC and Andrulis IL (1996) Heteroduplex and protein truncation analysis of the BRCA1 185delAG mutation. *Hum Genet* **98**: 310–312

Ozcelik H, Schmocker B, Di Nicola N, Shi XH, Langer B, Moore M, Taylor BR, Narod SA, Darlington G, Andrulis IL, Gallinger S and Redston M (1997) Germline BRCA2 6174delT mutations in Ashkenazi Jewish pancreatic cancer patients. *Nat Genet* **16**: 17–18

Phelan CM, Lancaster JM, Tonin P, Gumbs C, Cochran C, Carter R, Ghadirian P, Perret C, Moslehi R, Dion F, Faucher MC, Dole K, Karimi S, Foulkes W, Lounis H, Warner E, Goss P, Anderson D, Larsson C, Narod SA and Futreal PA (1996) Mutation analysis of the BRCA2 gene in 49 site-specific breast cancer families. *Nat Genet* **13**: 120–122

Simard J, Tonin P, Durocher F, Morgan K, Rommens J, Gingras S, Samson C, Leblanc JF, Belanger C, Dion F, Liu Q, Skolnick M, Goldgar D, Shattuck-Eidens D, Labrie F and Narod SA (1994) Common origins of BRCA1 mutations in Canadian breast and ovarian cancer families. *Nat Genet* **8**: 392–398

Southey MC, Tesoriero AA, Andersen CR, Jennings KM, Brown SM, Dite GS, Jenkins MA, Osborne RH, Maskiell JA, Porter L, Giles GG, McCredie MR, Hopper JL and Venter DJ (1999) BRCA1 mutations and other sequence variants in a population-based sample of Australian women with breast cancer. *Br J Cancer* **79**(1): 34–39

Szabo CI and King MC (1997) Population genetics of BRCA1 and BRCA2. *Am J Hum Genet* **60**: 1013–1020

Tavtigian SV, Simard J, Rommens J, Couch F, Shattuck-Eidens D, Neuhausen S, Merajver S, Thorlacius S, Offit K, Stoppa-Lyonnet D, Belanger C, Bell R, Berry S, Bogden R, Chen Q, Davis T, Dumont M, Frye C, Hattier T, Jammulapati S, Janecki T, Jiang P, Kehrer R, Leblanc JF, Leblane JF, Mitchell JT, MeArthur-Morrison J, Nguyenk K, Peng Y, Sanson C, Schroeder M, Snyder SC, Steele L, Stringfellow M, Stroup C, Swedlund B, Swensen J, Teng D, Thomas A, Tran A, Tran T, Tranchant M, Weaver-Feldhaus J, Wong AKC, Shizuya H, Eyfjord JE, Cannon-Albright L, Labrie F, Skolnick MH, Weber B, Kamb A and Goldgar DE (1996) The complete BRCA2 gene and mutations in chromosome 13q-linked kindreds. *Nat Genet* **12**: 333–337

Tadmouri GO, Tuzmen S, Ozcelik H, Ozer A, Baig SM, Senga EB and Basak AN (1998) Molecular and population genetic analyses of beta-thalassemia in Turkey. *Am J Hematol* **57**(3): 215–220

Turkish Ministry of Health (1994) Republic of Turkey Health Statistics, Turkish Ministry of Health (web page: www.saglik.gov.tr/turkce/istatistikler/s91_tur.htm)

Wooster R, Bignell G, Lancaster J, Swift S, Seal S, Mangion J, Collins N, Gregory S, Gumbs C, Micklem G, Barfoot M, Hamoudi R, Patel S, Rice C, Biggs P, Hashim Y, Smith A, Connor F, Arason A, Gudmundsson J, Ficenec D, Keisell D, Ford D, Tonin P, Bishop DT, Spurr NK, Ponder BAJ, Eeles R, Pete J, Devilee P, Carnelisse C, Lynch H, Narod S, Lenoir G, Eglisson V, Bjork-Barkadottir R, Easton DF, Bentley DR, Futreau PA, AshWorth A and Stratton MR (1995) Identification of the breast cancer susceptibility gene BRCA2. *Nature* **378**: 789–792

*© 2000 Cancer Research Campaign*
